# Iron Oxide Magnetic Nanoparticles Synthesized by Laser Target Evaporation Method for the Needs of Cancer Immunotherapy

**DOI:** 10.3390/ma18092142

**Published:** 2025-05-06

**Authors:** Felix Blyakhman, Fedor Fadeyev, Alexander Safronov, Tatiana Terziyan, Ekaterina Burban, Tatyana Shklyar, Galina Kurlyandskaya

**Affiliations:** 1 Department of Biomedical Physics, Ural State Medical University, Ekaterinburg 620028, Russia; feliks.blyakhman@urfu.ru (F.B.); fdf79@mail.ru (F.F.); t.f.shkliar@urfu.ru (T.S.); 2 Institute of Natural Sciences and Mathematics, Ural Federal University, Ekaterinburg 620002, Russia; alexander.safronov@urfu.ru (A.S.); tatiana.terzian@urfu.ru (T.T.); e.a.mikhnevich@urfu.ru (E.B.); 3 Institute of Medical Cell Technologies, Ekaterinburg 620026, Russia; 4 Institute of Electrophysics UB RAS, Ekaterinburg 620016, Russia

**Keywords:** magnetic nanoparticles, laser target evaporation method, maghemite, physicochemical properties, colloidal stability, immunotherapy, monocyte-derived dendritic cells, cytotoxicity

## Abstract

Administration of monocyte-derived dendritic cells (moDCs) sensitized by cancer-associated antigens to the patient is applied to boost the T-cell mediated anti-tumor immune response. Loading moDCs with magnetic nanoparticles (MNPs) and controlling their migration to lymph nodes by an external magnetic field is a way to improve the effectiveness of immunotherapy. In this study, spherical MNPs of maghemite iron oxide with a diameter of about 14 nm were synthesized by laser target evaporation method (LTE) and examined in the context of their prospective use for the needs of moDCs immunotherapy. Characterization of the physicochemical properties of MNPs and their stabilization in physiological media, as well as the magnetic properties of MNPs in the suspensions were considered in detail. The cytotoxic effect of MNPs in growth medium on the human moDCs and MNPs uptake by the cells were also estimated. We show that up-taken MNPs and MNPs in growth medium demonstrated cytotoxic effect only at high concentrations. At the same time, at low concentrations MNPs up-taken by moDCs increased their viability causing the stimulation effect. The evaluation of the quantity of MNPs, up-taken by cells, is possible by magnetometry even for the smallest γ-Fe_2_O_3_ concentrations.

## 1. Introduction

Low dimensional magnetic system becomes the subject of intensive research and applications with the development of nanotechnology [1,2]. They become a basis of fundamental research and new applications [3,4]. Magnetic nanoparticles (MNPs) form a large sub-area of modern hot spots in nanosciences representing multidisciplinary research involving colloidal, analytical and inorganic chemistry, material science, physics of magnetic phenomenon, and some other disciplines. MNPs are widely implemented for various applications of modern biomedicine [5,6,7].

Among a number of technologies (physical, chemical, biological) for the synthesis of MNPs, the electrophysical laser target evaporation (LTE) method has demonstrated certain advantages in comparison with others [8]. First of all, it allows the production of the batch of MNPs up to 100 g, which is very important for obtaining sufficient volumes of standardized material for multiple biological experiments [9]. It is especially critical in view of the fact that biomedical applications in a majority of cases require synthesis of stabilized water-based suspensions [10,11]. Here, one can mention that LTE MNPs due to the technique of synthesis (condensation from the gas phase) show very high degree of sphericity proven by transmission electron microscopy, magnetic measurements of the primary magnetization curves, and ferromagnetic resonance data [8]. In part, this high degree of sphericity contributes to the success in creation of water-based suspensions of different types, based on air-dried LTE MNPs batch.

In addition, the “teranostics” approach, which means combination of the diagnostics and therapy in one complex medical action, becomes more and more important [12,13,14]. Implication of magnetic materials together with magnetic field sensors ensures non-invasive control over the therapeutic stage over the time of diagnostics and/or therapy [15,16,17].

From the viewpoint of biomedical applications, it is critically important that MNPs synthesized by the LTE method have good biocompatibility and low toxicity supported among other factors by the uniformity of phase composition [3,11,18]. Since 2011, authors were publishing research results of biological experiments with MNPs obtained by LTE electrophysical technique [19,20,21,22]. It allowed to obtain large batches of air-dried MNPs (up to 100 g) and stabilized suspensions (up to 100 mL as a single batch for concentrations up to 5 wt.%). Interestingly, some of these studies were on purpose using MNPs from suspension of the same batch for different biological systems. Therefore, the results obtained in these experiments related to interactions of different biological systems become more comparable, as such nanomaterial as air-dried iron oxide MNPs had no apparent difference from experiment to experiment, even more importantly.

In nanomaterials applications, the air-dried batch of MNPs, if it is sufficiently large, can be a basis for the synthesis of different nanofluids, depending on the stabilization route [8,9,10,11]. In that case, the results of the studies do not depend on the variation of the MNPs ensemble varying from batch to batch. Here, there are some examples of previous studies related to blood mononuclear leukocytes/peripheral blood leucocytes, mesenchymal stem cells, chondrocytes, human dermal fibroblasts, algal, Exophiala nigrum (black yeasts), and their mutant strain (red yeasts) including in vivo experiments related to regererative medicine [19,20,21,22,23,24].

This study focuses on the interaction of LTE-MNPs with dendritic cells for the needs of immunotherapy. Dendritic cells (DCs) are a heterogenic group of specialized antigen-presenting cells processing tumor-associated antigens and presenting their epitopes to T-cells, thereby inducing the anti-cancer immune response. Subsets of the dendritic cells may form cellular networks that regulate both innate and adaptive immune responses. The immunostimulatory capacity of DCs makes them one of the central tools for cancer immunotherapy. Most commonly, the DCs derived from peripheral blood monocytes (moDCs) by cytokine exposure are used for immunotherapy [25].

The efficiency of moDC therapy can be increased by using moDCs with uptaken magnetic nanoparticles [26], which opened the prospects for seeking new approaches for moDC-based vaccines development. In particular, MNPs were proposed to be used as carriers to deliver tumor antigens (or mRNAs) to cytoplasm of dendritic cells [26,27]. Loading moDCs with MNPs allowed to track their distribution in organisms after administration to the patient by MRI [27,28,29].

Meanwhile, the moDCs therapy demonstrates limited efficacy due to imperfection of procedures of generation and administration of moDCs, resulting in their limited migration to lymph nodes [30]. It was demonstrated that after intradermal injections, most of moDCs left near the injection site, and less than 5% of them reached lymph nodes [31,32]. At the same time, the administration of anti-tumor vaccines based on MNPs-loaded DCs to experimental animals and exposure to external magnetic field after injection provided the increased efficiency of tumor inhibition growth [29,33].

Monocyte-derived dendritic cells were less studied by the community in part of the stimulatory effect of MNPs on moDCs viability. In fact, this effect was studied for the case of pigs [34], as they are considered to be the most approximate model to humans. Donini et al. [35] investigated whether magnetic or biomimetic magnetic nanoparticles affect relevant activities of human monocytes and found that the nanoparticles were ingested by monocytes and DCs without altering their viability. However, the knowledge about the regulation of moDCs in the initiation of immune responses under inflammatory conditions is still insufficient.

The aim of the present study is to investigate the possibility for stabilized suspensions of LTE MNPs to be used in application to control moDCs migration using an external magnetic field. At this stage of the study, the following tasks were chosen for solution: synthesizing of a large batch of LTE MNPs and stabilizing water-based suspension on their basis; characterization of physicochemical and magnetic properties of obtained materials; development of the techniques for reliable stabilization of MNPs in physiological media; assessing the possibility of MNPs internalization by moDCs, as well as estimation the cytotoxic effect of MNPs on the viability of moDCs.

## 2. Materials and Methods

### 2.1. Synthesis of Magnetic Nanoparticles and Their Colloidal Suspensions

Iron oxide magnetic nanoparticles (MNPs) were synthesized by laser target evaporation method using the apparatus and regimes described in refs [8,22]. The laboratory setup with Ytterbium (Yb) fiber laser with 1.07 mm wavelength was elaborated. The cylindrical target 65 mm in diameter and 20 mm in height was prepared by compaction of commercial magnetite microparticles (Alfa Aesar, Ward Hill, MA, USA). Evaporation was performed by a pulsed laser beam with focal spot 0.45 mm and 212 W output power. Condensation of particles took place in a gas mixture of N_2_ and O_2_ in the volume ratio 0.79:0.21. Transmission electron microscopy (TEM) images of MNPs were obtained using JEOL JEM2100 microscope (JEOL Corp., Tokyo, Japan) operated at 200 kV.

The X-ray diffraction characterization (XRD) was performed using D8 DISCOVER diffractometer (Bruker Corp., Billerica, MA, USA) operated at 40 kV at Cu-K*a* radiation (wavelength *l* = 1.5418 Å) with a graphite monochromator and scintillation detector. Bruker software TOPAS-3, with Rietveld full-profile refinement, was used for the quantitative diffractogram analysis.

Colloidal suspensions of air-dried MNPs in 5 mM sodium citrate were disaggregated by ultrasound treatment for 30 min using Cole-Parmer CPX-750 processor (Cole-Parmer, Vernon Hills, IL, USA) operated at 250 W. Permanent cooling of the suspension was provided. The remained aggregates were eliminated by centrifuging at 8000 rpm for 5 min (Hermle Z383 centrifuge; Hermle AG, Gosheim, Germany). To provide colloidal stability of the suspension in physiological solutions, electrosteric stabilizer ammonium poly(methacrylate) brand mark Darvan CN (Vanderbilt Chemicals, Norwalk, CT, USA) was then added to the suspension in 0.5 wt.% concentration. The stock suspension was then equilibrated in thermostat at 90 °C for 60 min. The final concentration of iron oxide MNPs in the stock ferrofluid was 4.24 wt.%. It was added to the cell culture media in pre-calculated portions to provide the desired concentration of iron oxide MNPs. The hydrodynamic diameter and zeta potential of species in MNPs suspensions were measured by dynamic light scattering (DLS) and electrophoretic light scattering (ELS) using Brookhaven ZetaPlus analyzer (Brookhaven Instruments, Holtsville, NY, USA). Colloidal stability in physiological solutions was tested by DLS and ELS at dilute suspensions with 0.01% concentration of MNPs.

### 2.2. Magnetic Measurements

Magnetic hysteresis loops or the dependences of magnetization M on the extremal magnetic field strength were measured for both as-prepared air-dried MNPs and MNPs obtained by drying the stabilized suspension. As our research line is focused on the possible contribution of MNPs to the maturation of DCs and their ability to migrate to lymph nodes, the average magnetic moment per single MNP in the suspension became an important parameter. It depends on the average size of the MNPs of ensemble [36,37] and appears to be smaller in comparison with as-prepared air-dried MNPs of the same batch due to the fact that larger MNPs are usually excluded from the ensemble during suspension preparation.

Samples for magnetic characterization were prepared as follows. The certain amount of either as-prepared air-dried MNPs or MNPs obtained by drying of stabilized suspension with concentration of 4.24 wt.% were placed into polycarbonate capsule. The field dependences of the MNPs material magnetization were measured using an MPMS-XL7 EC equipment (Quantum Design, San Diego, CA, USA) operating with a converter based on a SQUID (Superconducting Quantum Interference Device). The magnetic field H_max_ created by the solenoid was as high as ±70 kOe. In low fields range of ±5 kOe, magnetic fields could be varied with very fine steps of 0.1 Oe. In the other magnetic field ranges, the variation with a step of 1 Oe was used. Therefore, the measurement accuracy of the magnetic moments of the MNP’s samples and the permissible relative error in specifying the magnetic field strength were ±1.0%.

### 2.3. Cells Preparation and Measurements

#### 2.3.1. Generation of Human Monocyte-Derived Dendritic Cells

Peripheral blood mononuclear cells (PBMC) were isolated from blood of four healthy donors who signed appropriate informed voluntary consent to take part in this investigation. The Ethics Committee of the Institute of Medical Cell Technologies (Ekaterinburg, Russia) approved the study (protocol #8, 30 November 2021).

PBMCs were separated by density-gradient centrifugation using Lympholyte-H (Cedar Line, Burlington, ON, Canada) according to manufacturer’s instructions. PBMCs were collected, washed twice with Dulbecco’s phosphate buffer saline (DBPS) without Ca^2+^ and Mg^2+^ (Biolot, Saint-Petersburg, Russia) and resuspended in RPMI-1640 (PanEco, Moscow, Russia). Cells in suspension were seeded on the tissue-culture (TC) treated polysterene surface (in culture flasks or plates) and incubated for 2 h in CO_2_-incubator (37 °C, 5% CO_2_, humidified atmosphere) (MCO-15AC, Moriguchi, Osaka, Sanyo, Japan). After incubation, non-adherent cells (predominantly lymphocytes) were washed away by Earle’s balanced salt solution (EBSS) (PanEco, Moscow, Russia), while monocytes remained adhered on plastic. Monocytes were cultured in AIM-V medium (Gibco, Thermo FS, Waltham, MA, USA), supplemented with granulocyte-macrophage colony-stimulating factor (GM-CSF, 80 ng/mL) and interleukin 4 (IL-4, 25 ng/mL) (Sci-Store, Moscow, Russia) for stimulation of differentiation to immature monocyte-derived dendritic cells (moDCs). Cells were stimulated for 4 days: on the 2nd day, fresh portions of GM-CSF and IL-4 were added in the same concentrations. Differentiated cells had the common for immature moDCs elongated shape with processes. After stimulation, the immunophenotype of obtained moDCs was confirmed by flow cytometry [38,39,40].

#### 2.3.2. Flow Cytometry

MoDCs were detached from plastic by TrypLE (Gibco, Thermo FS, Waltham, MA, USA), cells remaining adhered were scrapped by cell scrapper. Cells were washed from TrypLE by phosphate buffered saline (PBS) and stained with fluorescent conjugated antibodies. The following antibodies were used: CD14-APC, CD11c-APC-Cy7, CD86-PC5.5, CD83-PC5.5, and HLA-DR-BV421 (Biolegend, San-Diego, CA, USA). The percentage of viable cells was measured by staining with Zombie Aqua (Biolegend).

#### 2.3.3. Prussian Blue Staining

MoDCs with MNPs adhered to the plastic surface were washed 3 times with PBS, fixed with 2.5% glutaric aldehyde, and dehydrated with ethanol. Cells were covered with staining solution, containing 2% of hydrochloric acid and 2% of potassium ferrocyanide (K_4_[Fe(CN)_6_]), and incubated for 15 min. After that, the staining solution was removed, and cells were washed with distilled water. Iron-containing MNPs provided the cells with the blue staining.

#### 2.3.4. MNPs Cytotoxicity Test

Cytotoxicity of MNPs was estimated by MTT-test. This widely introduced approach is a colorimetric test of biochemical activity of cells based on the reduction of a yellow tetrazolium salt (3-(4,5-dimethylthiazol-2-yl)-2,5-diphenyltetrazolium bromide) by cellular enzymes.

The test was conducted in wells of 48-well culture plates with TC treated surface. To decrease the mechanical injury of cells by nanoparticles, 10% fetal bovine serum (FBS) (PanEco, Moscow, Russia) was added to differentiation medium. The test was performed in two conditions: moDCs with up-taken MNPs in medium free of nanoparticles (i) and moDCs in medium with nanoparticles (ii).

Condition I. PBMC suspension in RPMI-1640 was seeded in wells of 48-well plates. MNPs were added immediately after dispensing cell suspension. Plates were incubated 3 h in the CO_2_-incubator. Non-adhered cells and unbound MNPs were removed by washing wells 3 times with EBSS; monocytes with up-taken MNPs remained adhered to plastic. After rinsing, the differentiation medium with FBS was dispensed into plate wells.

Condition II. After cell suspension dispensing in wells, plates were incubated for 3 h, non-adhered cells were washed away with EBSS. Differentiation medium with FBS was poured into plate wells, after that MNPs in appropriate concentrations were added.

For both conditions, the seeding density was 1–2 × 10^6^ cells/cm^2^ of plastic surface. As the MNPs gradually deposited from suspension on cell monolayer surface, their concentration is expressed in µg/cm^2^. MNPs were added in the final concentrations from 1 to 256 µg/cm^2^ (from 4 to 1024 µg/cm^3^, respectively). After cells seeding and MNPs addition, monocytes were differentiating to moDCs for 4 days. On the 2nd day, fresh cytokines were added to differentiation medium in all wells without medium change. After 4 days, the viability of obtained moDCs was measured using the MTT-test.

Differentiation medium was removed, cells were carefully washed with EBSS (PanEco, Moscow, Russia), and incubated with MTT solution (1 µg/mL, 100 µL per well) (Acros Organics, Thermo Fisher Scientific, Inc., Waltham, MA, USA) for 3 h in the CO_2_-incubator. MTT solution was removed, formazan crystals were dissolved in DMSO (200 µL per well), and 150 µL of formazan solution in DMSO from each well were transferred to new 96-well plates. This step allowed the exclusion of the optical density of MNPs in cells. The optical density of the solution was measured by a microplate reader (iMark, Bio-Rad, Hercules, CA, USA) with 490 nm working wavelength and 750 nm reference wavelength.

## 3. Results and Discussion

### 3.1. Characterization of Physicochemical Properties of MNPs and Suspensions

**Figure 1:**
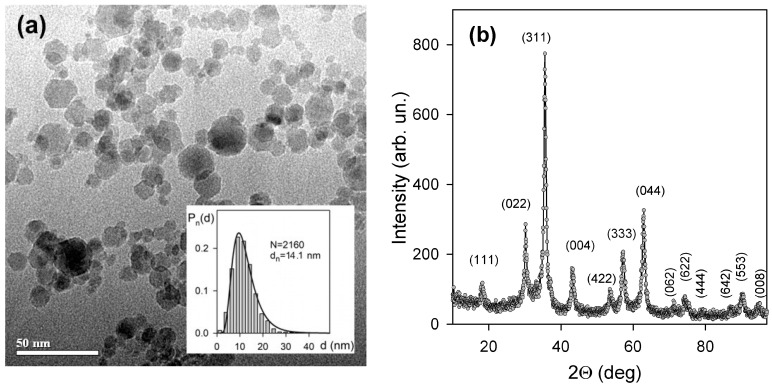
(**a**) TEM images of LTE MNPs. Insert: histograms of the particle size distributions (PSDs) lines give fitting of PSD with lognormal distribution function (median diameter 11.4 nm, logarithmic dispersion 0.42). (**b**) XRD pattern of MNPs crystalline structure.

Figure 1a presents the shape and characteristic dimensions of synthesized MNPs. Their shape was close to spherical. Several particle images show hexagonal faces. Particle size distribution (PSD) was calculated based on the graphical analysis of 2160 particle images. It was found lognormal with median 11.4 nm and logarithmic dispersion 0.42.

According to the XRD (Figure 1b) crystalline structure of iron oxide, MNPs corresponded to maghemite γ-Fe_2_O_3,_ which is typical for this sort of MNPs. Detailed discussion on the crystalline structure of LTE iron oxide MNPs can be found elsewhere [8,23].

Application of MNPs suspensions for the purposes of biomedicine and bioengineering depends on their stability in physiological and biological media. Concerning the objectives of the present study, at least two aspects of stability are to be considered. The first question is the stability of MNPs to aggregation and sedimentation in the standard physiological solutions which are elaborated for cell culturing. The second question is the stability of MNPs suspension up-taken by cell, though it can hardly be controlled or even monitored with certainty.

The first step of the present study was the estimation of the stability of MNPs solution. As it was discussed in our previous reports [8], spherical maghemite nanoparticles synthesized by the LTE method could be successfully dispersed in water due to electrostatic stabilization mechanism. It might be self-stabilization provided by the dissociation of traces of iron nitrates at the surface of MNPs, which are typically synthesized at high temperatures of laser evaporation of the oxide. Self-stabilization leads to positive values of zeta-potential ca. +30 mV due to the excess of Fe^+3^ ions at the surface. However, self-stabilization is sensitive to the presence of ions even in very low concentrations [8]. Stabilization with the use of sodium citrate as an electrostatic stabilizer is more reliable. In this case, MNPs in suspension get negatively charged due to the adsorption of citrate ions on their surfaces which provides zeta-potential ca. −40 mV. The threshold of stability to aggregation for citrate-stabilized LTE maghemite suspensions is ca. 0.1M NaCl, while in the case of self-stabilized suspensions, it is two orders of magnitude lower.

However, citrate stabilization of MNPs is still not sufficient in typical physiological solutions as the concentration of ions there is higher than the threshold of stability. In the present study, we have tested citrate-stabilized MNPs suspension with average hydrodynamic diameter 74 nm and zeta-potential −44.3 ± 6.4 mV. If placed in standard DMEM (Dulbecco’s Modified Eagle Medium) solution, the hydrodynamic diameter of the citrate-stabilized suspension would have jumped to 1240 nm. Zeta-potential would have dropped to −16.3 ± 2.9 mV. It resulted in rapid aggregation and sedimentation of MNPs. The reason for the loss of colloidal stability is the contraction of the double electrical layer on the surface of particles due to high ionic concentration in solutions and the screening of the electrostatic repulsion of MNPs.

Therefore, to provide the colloidal stability of MNPs suspension in physiological conditions, steric stabilization not sensitive to the ionic force of the solution needs to be additionally implemented.

**Figure 2:**
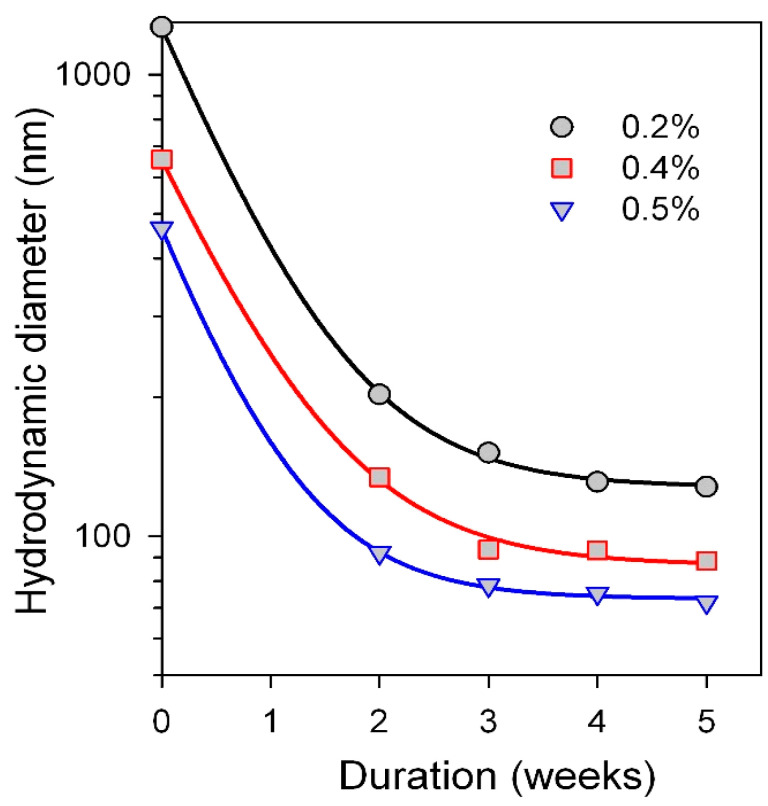
Mean average hydrodynamic diameter of species in MNPs suspension stabilized sequentially by sodium citrate and sodium poly(methacrylate) (Darvan CN) and placed in DMEM solution. Dependence on the duration of the preliminary storage of the suspension at 25 °C is given. Plots correspond to different concentrations of Darvan CN which are given in the legend. Lines are drawn as eye-guide only. Vertical axis is presented in logarithmic scale.

To maintain stability of the suspension of MNPs in physiological solutions, we have used a two-step protocol [41]. At the first step, aqueous suspension of MNPs was dispersed and disaggregated in the presence of electrostatic stabilizer 5 mM sodium citrate, as described in our earlier works [8]. Then, an electrosteric stabilizer—ammonium poly(methacrylate) (Darvan CN) was introduced in the system. The stability of the stock suspension in physiological conditions was tested periodically during its storage at room temperature. The probes of the stock suspension were mixed with the DMEM solution, and the mean average hydrodynamic diameter of species was monitored over time using DLS. Figure 2 shows that the stability of MNPs in DMEM solution substantially improved with the duration of the preliminary storage of stock suspension at room temperature.

One can see that as-prepared fresh suspension was not stable in DMEM solution. The mean average hydrodynamic diameter varied from 400 to 1200 nm depending on the concentration of Darvan CN. However, after the two-week preliminary storage of the stock suspension, the hydrodynamic diameter of MNPs introduced in DMEM solution decreased to 100–200 nm. The diminishing trend preserved upon further preliminary storage and after a 5 week period achieved saturation at 80–110 nm level.

The stabilization efficiency depended on the concentration of electrosteric stabilizer Darvan CN. The final average hydrodynamic diameter of MNP species in DMEM suspension was 129, 87, and 73 nm for Darvan CN concentrations 0.2%, 0.4%, and 0.5%, respectively. The latter value (73 nm) within the limits of experimental error was the same as the value of the hydrodynamic diameter in the stock suspension of MNPs stabilized by citrate in water. Therefore, Darvan CN concentration 0.5% was taken for the usage in the two-step protocol for MNPs stabilization in physiological solutions.

The data presented in Figure 2 indicates that the stability of MNPs in DMEM solution does not instantly appear if sodium citrate and Darvan CN are sequentially added to the stock suspension, but it takes substantial period of time for stabilization. In our opinion, the role of citrate is to provide initial dispersion and stabilization of MNPs. Due to its small molecular dimensions, citrate is much more efficient at this step than long polymeric anions of Darvan CN. When Darvan CN is added thereafter to the suspension already stabilized by citrate, it opens a competition between two stabilizers. Both are anionic and, in general, both adhere to the same centers of the surface of MNPs. At the beginning, these centers are occupied by citrate. But Darvan CN polyanions can replace citrate on the nanoparticles surface, and so, an equilibrium of the competitive adsorption should be established eventually. The ratio citrate/Darvan CN at the surface depends both on their relative concentration in the solution and on their relative adherence to the surface. The concentration of citrate was 5 mM (0.2%), that was rather close to Darvan CN concentration, 0.2–0.5%. The relative adherence of citrate/Darvan CN cannot be clarified with certainty for now. Meanwhile, we suppose that there might be an advantage in sorption of polymethacrylic anions of Darvan CN due to the multiple carboxylic residues in their polymeric chains. Therefore, we may assume that polyanions of Darvan CN can gradually substitute a major part of citrate anions at the surface of MNPs.

Thus, we suppose, that during the preliminary storage of the stock suspension, the electrostatic stabilizer (citrate) was substituted by electrosteric stabilizer—poly(methacrylate) Darvan CN. As a result, due to the polymeric layer adsorbed at the surface, the stability of MNPs in DMEM solution substantially improved. The higher the concentration of Darvan CN in the stock suspension, the higher the final stability of MNPs. Most likely, it indicates that at higher concentration of Darvan CN, substitution of citrate at the surface increased.

For now, however, we may only present the experimental evidence for the efficiency of the two-step protocol in the stabilization of iron oxide MNPs in physiological solutions. The mechanism remains hypothetical and needs clarification in special extended studies which we aim to perform in the future.

**Figure 3:**
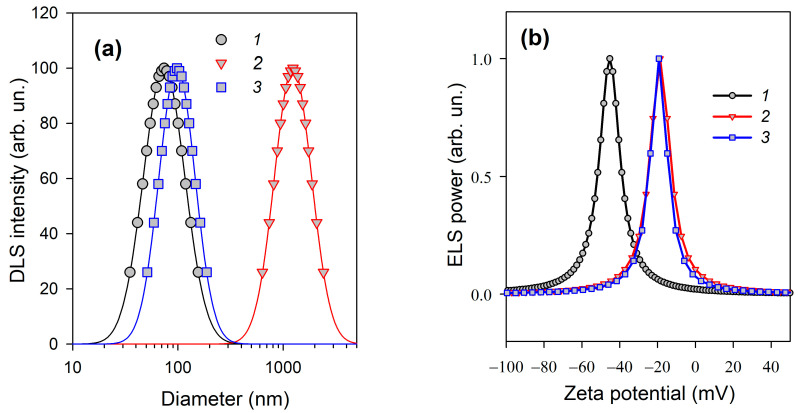
(**a**) Particle size distributions obtained via DLS in unimodal regime. Horizontal axis is presented in logarithmic scale. (**b**) Zeta potential of suspensions obtained via ELS. *1*—MNPs in the aqueous suspension stabilized by citrate; *2*—MNPs in DMEM stabilized by citrate; *3*—MNPs in DMEM stabilized by Darvan CN (0.5 wt.%) under two-step protocol with equilibration at 90 °C.

It is, certainly, practically inconvenient to store suspension of MNPs for several weeks preliminary to its usage. To avoid this, the kinetics of re-adsorption can be speeded up at elevated temperatures. We have found out that heating of the stock suspension of MNPs at 90 °C for 60 min provided the same effect on stability in DMEM as the long-term storage at room temperature. Figure 3a presents the particle size distributions (PSD) obtained by DLS in unimodal regime for MNPs in the aqueous suspension stabilized by citrate, for MNPs in DMEM stabilized by citrate, and for MNPs in DMEM stabilized by Darvan CN under two-step protocol with equilibration at 90 °C.

It can be seen that citrate-stabilized MNPs strongly aggregate in DMEM as the PSD shifts to values by an order of magnitude higher. At the same time, PSD for MNPs in DMEM stabilized by Darvan CN under two-step protocol remains almost the same as for MNPs in water. Along with the shift of the PSDs, their width also strongly changed during aggregation. It is not visually evident in Figure 3a as the axis of diameters has been logarithmically scaled. The numerical estimation of the width of the PSDs at half-height gave 83 nm for MNPs stabilized by citrate in water, 93 nm for MNPs stabilized by two-step protocol in DMEM, and 1220 nm for MNPs stabilized by citrate strongly aggregated in DMEM.

It is worth noting that zeta-potential for MNPs in DMEM was the same both for citrate-stabilized and for Darvan CN-stabilized particles (Figure 3b). In the former case, it was −16.3 ± 2.9 MB as given above, in the latter case it was −16.4 ± 3.0 mV. Such equality is reasonable as both stabilizers are anionic, both occupy the same centers on the surface of MNPs, and the activity of both is equally depressed by the ionic force in DMEM solutions. And both were substantially shifted lower to lower absolute values compared to zeta-potential of MNPs stabilized by citrate (Figure 3b). As it is known, absolute values of zeta-potential lower than 20 mV cannot provide efficient electrostatic stabilization of colloids. Thus, the effectiveness of stabilization of MNPs by Darvan CN in DMEM can be solely attributed to the barrier polymeric layer at the surface.

### 3.2. Magnetic Properties of MNPs

**Figure 4:**
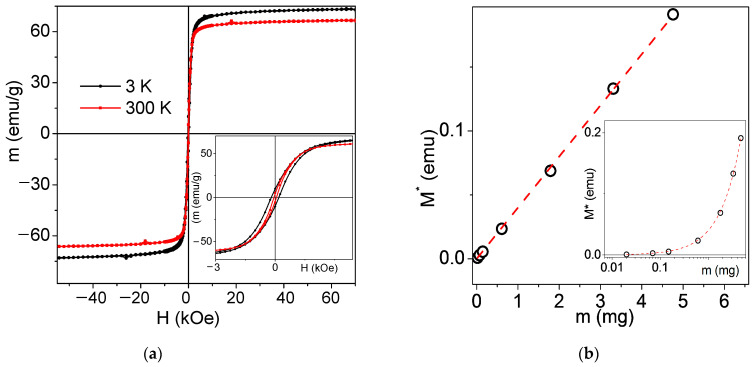
(**a**) Magnetic hysteresis loops of as-prepared air-dried MNPs measured at two different temperatures. Inset shows the same M(H) loops at low field range allowing estimation of the coercivity. Red arrow indicates the value of M(H = 70 kOe) at room temperature. (**b**) Magnetic moment M* corresponding to the sample of certain mass of MNPs measured in the external magnetic field H = 70 kOe at room temperature: points are the experimental data, and dashed line is the linear fit (R-Square = 0.9998). Inset shows the same data on a logarithmic scale allowing us to appreciate the accuracy of the measurements of very small amounts of the MNPs: black circles are the experimental data and dotted line is the exponential fit.

Figure 4a shows magnetic hysteresis loops of as-prepared air-dried MNPs measured at the temperatures of 3 and 300 K. Both the value of the saturation magnetization Ms (for simplicity approximated by the magnetization value M(H = 70 kOe)) and coercivity Hc are consistent with the average size of the ensemble of spherical MNPs of this type [42]. At low temperatures of 3 K, the coercive force is approximately three times higher, than at room temperature indicating the presence of a large number of MNPs in the state close to the superparamagnetic. Biomedical applications stimulated the development of different types of nanomaterials with nearly zero coercivity, i.e., close to zero magnetic moments in zero external magnetic fields [43,44]. Magnetic materials with nanosized elements (MNPs, nanowires, nanodisks) having non-zero magnetic moments in close to zero applied magnetic fields have tendency to aggregation due to magnetic interactions. They, therefore, are less suitable for many biomedical applications requiring thorough disaggregation for synthesis of stable water-based suspension [45] or usage of the naturally covered phospholipid membrane magnetite [46].

Figure 4b shows the summary of the measurements of different amounts of MNPs from dried stabilized suspension. Each point corresponds to the value of the magnetic moment of the sample of certain mass (M*) measured in the external magnetic field H = 70 kOe, i.e., external field sufficient for magnetic saturation. The first observation comes from the estimation of Ms value for MNPs of dried suspension: it is equal to approximately 39.4 emu/g at the room temperature. This value differs from the Ms 65 emu/g obtained in the case of air-dried MNPs. However, this difference is understandable because during the process of the synthesis of the suspension, the small fraction becomes stabilized and the process of interaction contributes to the Ms level to a lesser extent. In addition, one should take into account that about 2% of the mass of MNPs dried from suspension corresponds to the electrostatic stabilizer (means that Ms for MNPs of dried suspension is close to approximately 40.1).

The second observation is related to the minimum number of MNPs detectable by means of magnetic measurements. The lowest experimentally measured mass of MNPs was 2 × 10^−5^ g (Figure 4b). At the same time, the quality of the linear fit (R-Square = 0.9998) allows to make reasonable supposition that real detection limit of the employed technique is about 2–3 times lower. The minimum amount of MNPs used for biological experiments with MNPs in the present work was 2 μg/cm^2^ and the area of the well was as high as 0.83 cm^2^. This means the mass of MNPs was as high as 1.66 × 10^−6^ g per well or about an order of magnitude less in comparison with the limit of magnetic measurements. The evaluation of the quantity of up-taken MNPs using magnetic measurements is possible even for smallest concentrations of the order of 2 μg/cm^2^. Larger concentrations can be studied using a significantly smaller number of cells.

### 3.3. Cytotoxic Effects of MNPs Concentration on moDCs Viability and Estimations of the Magnetic Measurements Limits

**Figure 5:**
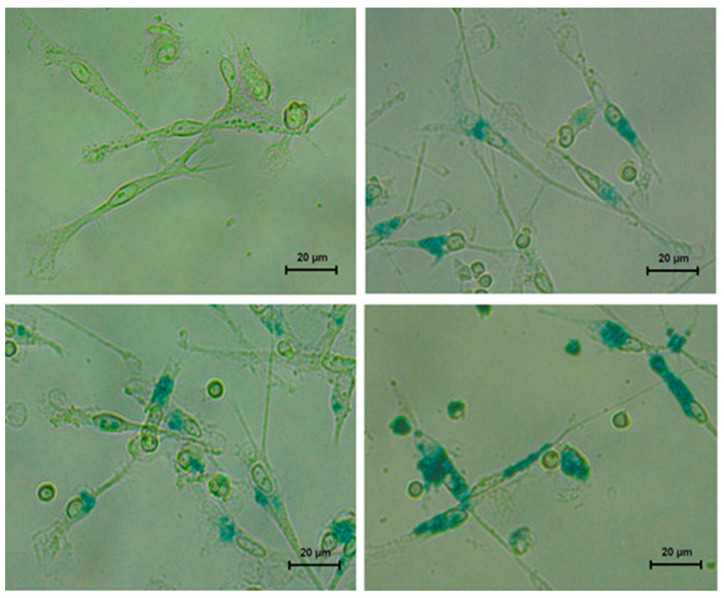
Prussian blue staining of moDCs with up-taken MNPs. Mononuclear cells were incubated with nanoparticles for 3 h, then non-adhered cells and MNPs were washed out. Remaining monocytes with up-taken MNPs were differentiated to moDCs under cytokine stimulation. The obtained moDCs were fixed and stained. Top row from left to right—the concentration of MNPs 0 and 2 μg/cm^2^, correspondingly; bottom row—8 and 64 μg/cm^2^.

The moDCs, differentiated from monocytes, had the typical elongated shape with multiple cytoplasmic processes (Figure 5, top row on the left). The DCs immunophenotype was confirmed by flow cytometry. Cells were gated according to the following scheme: primary selection of events based on forward/side scatter → doublet exclusion → exclusion of non-viable cells (stained by Zombie Aqua) → selection of CD11c+ cells. Gated cells demonstrated immunophenotype typical for immature moDCs: HLA-DR+ cells > 90%, CD86+ > 40%, CD40+ > 80%, CD14+ < 6%, and CD83 + <8% [38,39,40].

The toxicity assessment experiments were conducted in two conditions: (i) differentiation of monocytes to moDCs with up-taken nanoparticles in MNPs-free differentiation medium and (ii) differentiation of monocytes in medium containing MNPs, to distinguish the effect of extracellular and up-taken MNPs. The interaction of MNPs with moDCs in both experimental conditions was confirmed by Prussian blue staining. The intensity of blue coloring of cells demonstrated the dependence of the quantity of up-taken nanoparticles from their concentration (Figure 5).

Using the optical microscopy images, a rough estimation of the number of cells per unit square for all concentrations studied is possible. As the surface area corresponding to one well is 0.83 cm^2^, one can get an estimation (Figure 5, top row on the right) of about 3.9 × 10^4^ cells/cm^2^ or 3.2 × 10^4^ cells/well. The number of MNPs per one well for 2 μg/cm^2^ can be estimated by taking into account the density of γ-Fe_2_O_3_ (4.9 g/cm^3^). One can calculate the number of spherical MNPs with an average diameter about 14 nm in the 1.66 × 10^−6^ g batch of one well—it is about 2.4 × 10^11^ particles. This means 7.5 × 10^6^ MNPs/cell in the initial state, i.e., cultivation medium. Although cell morphology shows visible variations, just for the sake of very general estimation, one can calculate the average cell volume to be of the order of 2 × 10^−10^ cm^3^. Calculation of the total volume of all MNPs of the well for 2 μg/cm^2^ concentration shows that the volume of the grown cells is only 20 times larger than that of the volume of MNPs added to the cultivation medium, being plausible as the scenario with cells migration to lymph nodes seems to be realistic with such a density of packaging. Here, it is necessary to mention that not all MNPs added to the cultivation medium are accepted by cells. In addition, the presence of MNPs in cultivation medium may contribute to the cell’s development via ionic component, which is still quite an obscure subject in this area of research.

Estimation of the number of MNPs participating in the interaction with the cells can be done by counting the number of cells, and number of MNPs inside the cells using transmission electron microscopy [21] completed by the measurements of the magnetic properties of supernatants for known magnetic characteristics of the MNPs of stabilized suspension themselves. It can also be done by the measurements of magnetic properties of a carefully washed cell sample (for example for all cells collected from one well). The advantages of magnetic measurements of the cell culture containing MNPs are obvious. They are fast, simple and ensure that contributions of all particles are taken into account. At the same time, very small concentrations of MNPs and diamagnetic contribution of biological materials make some limitations or, better to say, require elaboration of the measurement procedure for each particular cell culture and batch of the MNPs. In addition, magnetic measurements can be useful as independent technique for the verification of the results obtained in the course of development of new methods, such as computer vision, for example. Recently, the first positive results on the application of computer vision algorithms for assessing the content of MNPs up-taken by dendritic cells in vitro were demonstrated [47].

Above, for the evaluation of the quantity of up-taken MNPs, it was concluded that the whole well-related sample should be analyzed for the smallest concentrations of the MNPs. However, it was also mentioned that (Figure 2) about twice smaller concentration evaluations are also reliable. In biological sciences, the statistics are very important; this conclusion about reliable comparison of the results of the cell growth experiments for the whole samples related to different wells even for very small MNPs concentrations seems to be very important.

The experimental data related to acquiring the location of the MNPs-loaded moDCs in space and estimation of their numbers are not provided here. It will be the next step of research. Previously, careful analysis of LTE MNPs location was done using transmission electron microscopy for the case of LTE MNPs water-based suspensions of two different types interacting with mononuclear leukocytes or human mesenchymal stem cells [48]. Suspensions with and without chitosan enhanced the secretion of cytokines by a 24 h culture of human blood mononuclear leukocytes compared to a control without MNPs. At 2.3 maximum permissive dose for chitosan stabilized suspension, MNPs presence promotes the stimulating effect on cells as observed in the present study. However, the goals of the present studies and studies with mononuclear leukocytes or human mesenchymal stem cells were different, and therefore, the importance of exact location of the MNPs is also different. As moDCs are expected to be employed in order to overcome their limited migration to lymph nodes by increasing the migration ability using external magnetic field, their exact location is not very important—the concentration per each cell is what matters.

In addition, this research has practical value. It is clear that one of the possible ways to increase the effectiveness of moDCs-based therapy is the direct guidance of cell migration to lymph nodes. It can be performed by a magnetic field after administration of the patient’s moDCs loaded with magnetic nanoparticles. Quantitative control of their position in space can be performed by the magnetic field sensor. Among others, the magnetoimpedance based detectors seem to be very promising as they can operate at room temperature and show the sensitivity up to 10^−8^ Oe level [7,49,50].

**Figure 6:**
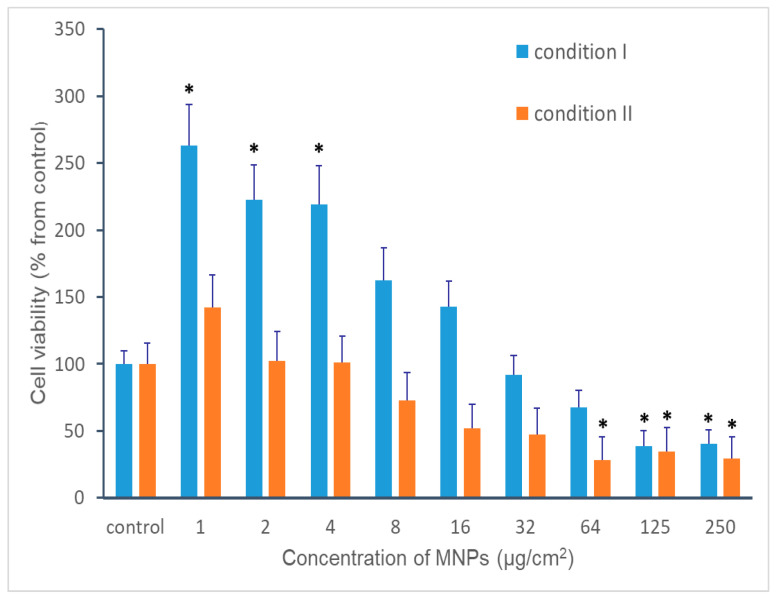
The cytotoxicity of MNPs to human moDCs at various concentrations of nanoparticles. Experiment was performed in two conditions to distinguish the effect of up-taken and extracellular nanoparticles. Condition I: moDCs with up-taken nanoparticles were incubated in MNPs-free medium; condition II: cells were incubated in medium containing MNPs. Cell viability was measured by MTT-test. Results of MTT-test were normalized to control without MNPs (accepted as 100%). Asterisks (*) indicate values that are statistically significant from control (*p* < 0.05).

The results of cytotoxicity test are shown in Figure 6. One can see that the MNPs demonstrate moderate cytotoxic effect: in both experimental conditions, statistically significant reducing of cell viability was observed only at high concentrations of nanoparticles. The cytotoxic effect was more pronounced when MNPs were present in differentiation medium (condition II). The IC50 for condition I and II were 21.94 ± 5.78 and 8.24 ± 2.58 µg/cm^2^, respectively. At low concentrations (1–8 μg/cm^2^), up-taken nanoparticles (condition I) even increase the moDCs biochemical activity. It is noteworthy, that increasing biochemical activity was not observed in the presence of MNPs in differentiation medium (condition II).

The deleterious effect of MNPs in high concentrations on various types of cells, including DCs [51,52], or the absence of impact of magnetic nanoparticles on DCs viability [28,35,53,54] was commonly described by other researchers. The cytotoxic effect of MNPs can be, at least partially, due to mechanical damage of cells by extracellular nanoparticles. This assumption is confirmed by the intensive detachment of moDCs after mixing the medium by pipette and by the higher cytotoxicity of extracellular MNPs compared to up-taken nanoparticles.

The effect of the increase of cell biochemical activity and proliferation at low concentrations of MNPs was observed in previous work with human fibroblasts [23]. The positive impact of MNPs on proliferation mesenchymal stromal cells (MSCs) was also described by other researchers [55]. At the same time, the stimulatory effect of MNPs on moDCs viability is not found in the literature.

The mechanism of positive impact of up-taken MNPs on cells’ biochemical activity/proliferation at low concentrations remains unclear. It cannot be excluded, that MNPs may be the extra source of ferrous ions for cells. Additionally, it was notified that MNPs can bind the TC-treated polystyrene surface, and the adhered MNPs are not removed by washing. It can also be suggested that adhered MNPs can facilitate the adhesion of cells. This suggestion can be indirectly confirmed by the differences in cell shape: moDCs with up-taken nanoparticles at low concentrations seemed to be more elongated and had longer processes in comparison with MNP-free control (see Figure 5).

Previously, Donini et al. [35] suggested that magnetic nanoparticles seem to be candidates for medical applications because they do not activate pro-inflammatory activities of monocytes, and their ability to stimulate DC maturation could be used for the vaccines design, as well as that harmlessly engulfed nanoparticles could be vehicles to carry molecules inside the immune cells for the immune response regulation. The present study of the behavior of moDCs uploaded by LTE-MNPs in a magnetic field is the subject of future planned research. However, magnetic measurements of model samples indicate the possibility of non-contact detection of the clusters of LTE MNPs either internalized or associated with cell membrane by magnetic field sensor having the external field sensitivity of the order of 1 nT.

## 4. Conclusions

It was suggested that the effectiveness of moDCs immunotherapy can be increased by loading cells with MNPs in order to control their position in space by an external magnetic field after administration, and to guide moDCs movement directly to lymph nodes. moDCs were obtained from blood of four healthy donors. Spherical γ-Fe_2_O_3_ magnetic nanoparticles of about 14 nm were synthesized by laser target evaporation method followed by the synthesis of stabilized suspension (citrate-stabilized MNPs suspension with average hydrodynamic diameter 74 nm and zeta-potential −44.3 ± 6.4 mV) for the needs of moDCs immunotherapy. Characterization of the physicochemical properties of MNPs and their two-step stabilization in physiological media showed a high level of colloidal stability.

Up-taken MNPs and MNPs in growth medium demonstrated cytotoxic effect only at high concentrations. Interestingly, at low concentrations, MNPs up-taken by moDCs increased their viability showing the stimulation effect.

The cytotoxic effect was more pronounced when MNPs were present in differentiation medium (condition II). The IC50 for condition I and II were 21.94 ± 5.78 and 8.24 ± 2.58 µg/cm^2^, respectively. At low concentrations (1–8 μg/cm^2^), up-taken nanoparticles (condition I) even increase the moDCs’ biochemical activity. The evaluation of the quantity of up-taken MNPs using even part of the individual well-related sample for the smallest γ-Fe_2_O_3_ MNPs amounts is possible by using magnetometry.

It is shown, that spherical LTE-MNPs of maghemite iron oxide, ~14 nm in average diameter, up-taken by the human moDCs, are quite suitable material for the creation of prospective magnetically controlled immunotherapy.

## Data Availability

The original contributions presented in this study are included in the article. Further inquiries can be directed to the corresponding author.

## Abbreviations

The following abbreviations are used in this manuscript:

moDCs	monocyte-derived dendritic cells
MNPs	magnetic nanoparticles
LTE	laser target evaporation
XRD	X-ray diffraction
TEM	Transmission electron microscopy
SQUID	Superconducting Quantum Interference Device
PSD	particle size distribution
DMEM	Dulbecco’s Modified Eagle Medium
PBMC	Peripheral blood mononuclear cells
DBPS	Dulbecco’s phosphate buffer saline
EBSS	Earle’s balanced salt solution

## References

[1] Pruna A., Poliac I., Busquets-Mataix D., Ruotolo A. (2025). Effect of electrodeposition conditions on adsorption and photocatalytic properties of ZnO. Materials.

[2] Freitas P.P., Ferreira R., Cardoso S. (2016). Spintronic sensors. Proc. IEEE.

[3] Zamani Kouhpanji M.R., Stadler B.J.H. (2020). Magnetic nanowires for quantitative detection of biopolymers. AIP Adv..

[4] Burks E.C., Gilbert D.A., Murray P.D., Flores C., Felter T.E., Charnvanichborikarn S., Kucheyev S.O., Colvin J.D., Yin G., Liu K. (2020). 3D nanomagnetism in low density interconnected nanowire networks. Nano Lett..

[5] Gloag L., Mehdipour M., Chen D., Tilley R.D., Gooding J.J. (2019). Advances in the application of magnetic nanoparticles for sensing. Adv. Mater..

[6] Beato J., Pérez-Landazábal J., Gómez-Polo C. (2018). Enhanced magnetic nanoparticle detection sensitivity in non-linear magnetoimpedance-based sensor. IEEE Sens. J..

[7] Barrera G., Celegato F., Vassallo M., Martella D., Coïsson M., Olivetti E.S., Martino L., Sözeri H., Manzin A., Tiberto P. (2024). Microfluidic detection of spions and co-ferrite ferrofluid using amorphous wire magneto-impedance sensor. Sensors.

[8] Safronov A.P., Beketov I.V., Komogortsev S.V., Kurlyandskaya G.V., Medvedev A.I., Leiman D.V., Larranaga A., Bhagat S.M. (2013). Spherical magnetic nanoparticles fabricated by laser target evaporation. AIP Adv..

[9] Grossman J.H., McNeil S.E. (2012). Nanotechnology in cancer medicine. Phys. Today.

[10] Pankhurst Q.A., Connolly J., Jones S.K., Dobson J. (2003). Applications of magnetic nanoparticles in biomedicine. J. Phys. D Appl. Phys..

[11] Ansari S.A.M.K., Ficiarà E., Ruffinatti F.A., Stura I., Argenziano M., Abollino O., Cavalli R., Guiot C., D’Agata F. (2019). Magnetic Iron Oxide Nanoparticles: Synthesis, Characterization and Functionalization for Biomedical Applications in the Central Nervous System. Materials.

[12] Pavlov A.M., De Geest B.G., Louage B., Lybaert L., De Koker S., Koudelka Z., Sapelkin A., Sukhorukov G.B. (2013). Magnetically engineered microcapsules as intracellular anchors for remote control over cellular mobility. Adv. Mater..

[13] Zverev V.I., Pyatakov A.P., Shtil A.A., Tishin A.M. (2018). Novel applications of magnetic materials and technologies for medicine. J. Magn. Magn. Mater..

[14] Wang T., Zhou Y., Lei C., Luo J., Xie S., Pu H. (2017). Magnetic impedance biosensor: A review. Biosens. Bioelectron..

[15] Llandro J., Palfreyman J.J., Ionescu A., Barnes C.H.W. (2010). Magnetic biosensor technologies for medical applications: A review. Med. Biol. Eng. Comput..

[16] Alsharif N.A., Martiinez-Banderas A., Merzaban J., Ravasi T., Kosel J. (2019). Biofunctionalizing magnetic nanowires toward targeting and killing leukemia cancer cells. IEEE Trans. Magn..

[17] Devkota J., Howell P., Mukherjee P., Srikanth H., Mohapatra S., Phan M.H. (2015). Magneto-reactance based detection of MnO nanoparticle-embedded Lewis lung carcinoma cells. J. Appl. Phys..

[18] Osipov V.V., Kotov Y.A., Ivanov M.G., Samatov O.M., Lisenkov V.V., Platonov V.V., Murzakaev A.M., Medvedev A.I., Azarkevich E.I. (2006). Laser synthesis of nanopowder. Laser Synth. Nanopowders.

[19] Blyakhman F.A., Makarova E.B., Shabadrov P.A., Fadeyev F.A., Shklyar T.F., Safronov A.P., Komogortsev S.V., Kurlyandskaya G.V. (2020). Magnetic nanoparticles as a strong contributor to the biocompatibility of ferrogels. Phys. Metals Metallogr..

[20] Blyakhman F.A., Melnikov G.Y., Makarova E.B., Fadeyev F.A., Sedneva-Lugovets D.V., Shabadrov P.A., Volchkov S.O., Mekhdieva K.R., Safronov A.P., Fernández Armas S. (2020). Effects of constant magnetic field to the proliferation rate of human fibroblasts grown onto different substrates: Tissue culture polystyrene, polyacrylamide hydrogel and ferrogels γ-Fe_2_O_3_ magnetic nanoparticles. Nanomaterials.

[21] Kurlyandskaya G.V., Novoselova I.P., Schupletsova V.V., Andrade R., Dunec N.A., Litvinova L.S., Safronov A.P., Yurova K.A., Kulesh N.A., Dzyuman A.N. (2017). Nanoparticles for magnetic biosensing systems. J. Magn. Magn. Mater..

[22] Fadeyev F.A., Blyakhman F.A., Safronov A.P., Melnikov G.Y., Nikanorova A.D., Novoselova I.P., Kurlyandskaya G.V. (2022). Biological impact of -Fe_2_O_3_ magnetic nanoparticles obtained by laser target evaporation: Focus on magnetic biosensor applications. Biosensors.

[23] Kulesh N.A., Novoselova I.P., Safronov A.P., Beketov I.V., Samatov O.M., Kurlyandskaya G.V., Morozova M.V., Denisova T.P. (2016). Total reflection X-ray fluorescence spectroscopy as a tool for evaluation of iron concentration in ferrofluids and yeast samples. J. Magn. Magn. Mater..

[24] Kurlyandskaya G.V., Safronov A.P., Shcherbinin S.V., Beketov I.V., Blyakhman F.A., Makarova E.B., Korch M.A., Svalov A.V. (2021). Magnetic nanoparticles obtained by electrophysical technique: Focus on biomedical applications. Phys. Solid State.

[25] Bol K.F., Schreibelt G., Rabold K., Wculek S.K., Schwarze J.K., Dzionek A., Teijeira A., Kandalaft L.E., Romero P., Coukos G. (2019). The clinical application of cancer immunotherapy based on naturally circulating dendritic cells. J. Immunother. Cancer.

[26] Huang L., Liu Z., Wu C., Lin J., Liu N. (2022). Magnetic nanoparticles enhance the cellular immune response of dendritic cell tumor vaccines by realizing the cytoplasmic delivery of tumor antigens. Bioeng. Transl. Med..

[27] Grippin A.J., Wummer B., Wildes T., Dyson K., Trivedi V., Yang C., Sebastian M., Mendez-Gomez H.R., Padala S., Grubb M. (2019). Dendritic cell-activating magnetic nanoparticles enable early prediction of antitumor response with magnetic resonance imaging. ACS Nano.

[28] de Vries I.J., Lesterhuis W.J., Barentsz J.O., Verdijk P., van Krieken J.H., Boerman O.C., Oyen W.J., Bonenkamp J.J., Boezeman J.B., Adema G.J. (2005). Magnetic resonance tracking of dendritic cells in melanoma patients for monitoring of cellular therapy. Nat. Biotechnol..

[29] Jin H., Qian Y., Dai Y., Qiao S., Huang C., Lu L., Luo Q., Chen J., Zhang Z. (2016). Magnetic enrichment of dendritic cell vaccine in lymph node with fluorescent-magnetic nanoparticles enhanced cancer immunotherapy. Theranostics.

[30] Gardner A., de Mingo Pulido Á., Ruffell B. (2020). Dendritic cells and their role in immunotherapy. Front. Immunol..

[31] Sabado R.L., Bhardwaj N. (2010). Directing dendritic cell immunotherapy towards successful cancer treatment. Immunotherapy.

[32] Morisaki T., Morisaki T., Kubo M., Morisaki S., Nakamura Y., Onishi H. (2022). Lymph nodes as anti-tumor immunotherapeutic tools: Intranodal-tumor-specific antigen-pulsed dendritic cell vaccine immunotherapy. Cancers.

[33] Khranovska N., Skachkova O., Gorbach O., Inomistova M., Orel V. (2021). Magnetically sensitive nanocomplex enhances antitumor efficacy of dendritic cell-based immunotherapy. Exp. Oncol..

[34] Qu X., Cinar M.U., Fan H., Pröll M., Tesfaye D., Tholen E., Looft C., Hölker M., Schellander K., Uddin M.J. (2015). Comparison of the innate immune responses of porcine monocyte-derived dendritic cells and splenic dendritic cells stimulated with LPS. Innate Immun..

[35] Donini M., Pettinella F., Zanella G., Gaglio S., Laudanna C., Jimenez-Carretero M., Jimenez-Lopez C., Perduca M., Dusi S. (2023). Effects of magnetic nanoparticles on the functional activity of human monocytes and dendritic cells. Int. J. Mol. Sci..

[36] Philip J. (2023). Magnetic nanofluids (Ferrofluids): Recent advances, applications, challenges, and future directions. Adv. Colloid Interface Sci..

[37] Jiao J., Zhang H., Zheng J. (2022). Ferrofluids transport in bioinspired nanochannels: Application to electrochemical biosensing with magnetic-controlled detection. Biosens. Bioelectron..

[38] Figueroa G., Parira T., Laverde A., Casteleiro G., El-Mabhouh A., Nair M., Agudelo M. (2016). Characterization of human monocyte-derived dendritic cells by imaging flow cytometry: A comparison between two monocyte isolation protocols. J. Vis. Exp..

[39] Mikloska Z., Bosnjak L., Cunningham A.L. (2001). Immature monocyte-derived dendritic cells are productively infected with herpes simplex virus type 1. J. Virol..

[40] Kim M., Kim J. (2019). Properties of immature and mature dendritic cells: Phenotype, morphology, phagocytosis, and migration. RSC Adv..

[41] Burban E.A., Fadeyev F.A., Safronov A.P., Blyakhman F.A., Terzian T.V., Neznakhin D.S., Yushkov A.A., Kurlyandskaya G.V. (2024). Determination of limits for evaluating the degree of internalization of γ-Fe_2_O_3_ nanoparticles by cultures of human mesenchymal stomal cells. Colloid J..

[42] Kurlyandskaya G.V., Portnov D.S., Beketov I.V., Larrañaga A., Safronov A.P., Orue I., Medvedev A.I., Chlenova A.A., Sanchez-Ilarduya M.B., Martinez-Amesti A. (2017). Nanostructured materials for magnetic biosensing. Biochim. Biophys. Acta (BBA) Gen. Subj..

[43] Castellanos-Rubio I., Insausti M., Garaio E., Gil De Muro I., Plazaola F., Rojo T., Lezama L. (2014). Fe_3_O_4_ nanoparticles prepared by the seeded-growth route for hyperthermia: Electron magnetic resonance as a key tool to evaluate size distribution in magnetic nanoparticles. Nanoscale.

[44] Goiriena-Goikoetxea M., García-Arribas A., Rouco M., Svalov A.V., Barandiaran J.M. (2016). High-yield fabrication of 60 nm Permalloy nanodiscs in well-defined magnetic vortex state for biomedical applications. Nanotechnology.

[45] Darton N.J., Ionescu A., Llandro J. (2019). Magnetic Nanoparticles in Biosensing and Medicine.

[46] De Cos D., Lete N., Fdez-Gubieda M.L., Garcia-Arribas A. (2020). Study of the influence of sensor permeability in the detection of a single magnetotactic bacterium. J. Magn. Magn. Mater..

[47] Mikushin P., Fadeyev F., Starodumov I., Bugayova A., Shklyar T., Blyakhman F. (2025). Application of computer vision algorithms for assessing the content of magnetic nanoparticles uptaken by dendritic cells in-vitro. IEEE Xplore.

[48] Kurlyandskaya G.V., Litvinova L.S., Safronov A.P., Schupletsova V.V., Tyukova I.S., Khaziakhmatova O.G., Slepchenko G.B., Yurova K.A., Cherempey E.G., Kulesh N.A. (2017). Water-based suspensions of iron oxide nanoparticles with electrostatic or steric stabilization by chitosan: Fabrication, characterization and biocompatibility. Sensors.

[49] Uchiyama T., Mohri K., Honkura Y., Panina L.V. (2012). Recent advances of pico-Tesla resolution magneto-impedance sensor based on amorphous wire CMOS IC MI sensor. IEEE Trans. Magn..

[50] Kurlyandskaya G.V., Buznikov N.A., Svalov A.V. (2024). Giant Magnetoimpedance: 30 years since rediscovery and next steps. Phys. Met. Metallogr..

[51] Zhang W., Zhang S., Xu W., Zhang M., Zhou Q., Chen W. (2017). The function and magnetic resonance imaging of immature dendritic cells under ultrasmall superparamagnetic iron oxide (USPIO)-labeling. Biotechnol. Lett..

[52] Goya G.F., Marcos-Campos I., Fernández-Pacheco R., Sáez B., Godino J., Asín L., Lambea J., Tabuenca P., Mayordomo J.I., Larrad L. (2008). Dendritic cell uptake of iron-based magnetic nanoparticles. Cell Biol. Int..

[53] Zhu R., Zhu Y., Zhang M., Xiao Y., Du X., Liu H., Wang S. (2014). The induction of maturation on dendritic cells by TiO_2_ and Fe(3)O(4)@TiO(2) nanoparticles via NF-κB signaling pathway. Mater. Sci. Eng. C Mater. Biol. Appl..

[54] Reddy L.H., Arias J.L., Nicolas J., Couvreur P. (2012). Magnetic Nanoparticles: Design and Characterization, Toxicity and Biocompatibility, Pharmaceutical and Biomedical Applications. Chem. Rev..

[55] Chang Y.K., Liu Y.P., Ho J.H., Hsu S.C., Lee O.K. (2012). Amine-surface-modified superparamagnetic iron oxide nanoparticles interfere with differentiation of human mesenchymal stem cells. J. Orthop. Res..

